# Difficult Diagnosis of Yellow Fever Cases*Read before the Louisiana State Medical Society, May 10, 1906, by L. Sexton, M. D., Medical Department Tulane University, Medical Director Louisiana National Life Society, etc.

**Published:** 1906-07

**Authors:** L. Sexton

**Affiliations:** New Orleans, La.


For Texas Medical Journal.

### Difficult Diagnosis of Yellow Fever Cases.*

BY L. SEXTON, M. D., NEW ORLEANS, LA.

The question of diagnosing cases of yellow fever in a given community after an epidemic has broken out, is comparatively easy, though doubtless many cases are improperly called yellow fever under the above named conditions, but the correct diagnosis of the first case of yellow fever that occurs in the spring, summer and fall months in a community where yellow fever has not previously been known to exist, is quite another proposition.

Acclimating, walking or mild cases of yellow fever, or cases occurring in children, and among colored people, are very difficult to diagnose.

Nearly all diseases have symptoms which, to the clinician, are known as cardinal symptoms. If you were asked for these cardinal symptoms in yellow fever, you would perhaps say — fever with jaundice, albuminuria following a chill after an acute onset, with tendency to hemorrhagic gums, and nauseated stomach with slow pulse, and great prostration following the fever.

The recent difference of opinion in regard to cases occurring this winter and spring, caused the President of your Section to ask

---

*Read before the Louisiana State Medical Society, May 10, 1906, by L. Sexton, M. D., Medical Department Tulane University, Medical Director Louisiana National Life Society, etc.

some one to prepare a paper on these differential points, and the lot fell to me.

I have nothing particularly new to offer in regard to the diagnosis of early cases of yellow fever, but a short review of some of the cardinal symptoms at this particular season and time may not be inopportune, and as a guide for future reference may help some of us when these close diagnoses, upon which so much depends, are to be made.

Given: In any tropical climate an acute febrile condition, following a chill and jaundice, stasis, albuminuria and disproportion between the pulse and temperature, excessive back, leg and head pains, with great prostration, how are we to honestly come to a conclusion with regard to the case, provided such be the first case, terminating by recovery?

In studying such a case we should, if possible, obtain the full history as to his habits and whereabouts for the previous three to five days before the onset. The initial chill in yellow fever is supposed to come on most often at night—many exceptions to this rule occur.

From such a beginning in this given case, after he had been in communication with some vessel, or sick person from some tropical climate, it would render the diagnosis of yellow fever very probable if stegomyia fasciata mosquitoes are present.

If, after the first twenty-four or thirty-six hours this temperature of 104° or 105° gradually falls to normal, with a slow pulse and great prostration, followed by perhaps a secondary rise of temperature after thirty-six or forty-eight hours, with albuminuria, and slight jaundice, the suspicion of yellow fever would be greatly increased; but no positive diagnosis could be made until malarial fever had been excluded by an absence of the plasmodium malaria in the blood. Not one, but several, examinations having been made. Typhoid should also be excluded by Widal reaction.

It would also become necessary to eliminate the possibility of a case of catarrhal jaundice, or albuminuria, and some fever resultant from auto-infection.

Severe back, head, loin and muscular pains; flushed face and hyperemic eyes; a congested skin and neck and chest; a sort of stasis of the blood with bleeding gums and narrow, red-edged tongue with middle coating; a slightly jaundiced appearance, becoming more marked each succeeding day; pulse becoming less frequent for three to four days, until forty-five or fifty-five beats per minute are recorded; highly colored, scanty albuminous urine, and finally hemorrhage from mucous membrane, suppression of the urine and black vomit with uremic coma—makes a very good pen picture of a case of yellow fever with perhaps a fatal termination.

Several of these cases occurring in the same family, or being traced to the same infected house, leave hardly a doubt of the nature and character of the disease, even though no epidemic should have been pronounced. But, it is the mild, non-albuminuric, or, in some, even walking cases of yellow fever, about which there is so often an honest difference of opinion. Many of these cases in our last three epidemics have not even gone to bed on account of the fear of quarantine or the inconvenience of fumigation incident to being reported sick.

The range of temperature in this mild yellow fever is from 99° to 101°—1000 of such cases occurring at Ocean Springs in 1897 without a death,—the real nature of the disease was not suspected until it attacked one whose constitution was already in a run-down condition, with resultant serious symptoms—black vomit, suppression, and death.

Many of these mild types of yellow fever go unrecognized and are accountable for the spread of disease in the strictly quarantined towns where the tracing of infection is otherwise impossible except upon the theory of these walking cases of yellow fever, bringing the infection into the town or city before the quarantine restrictions were put on, or the nature of the disease understood. It is such cases of mild yellow fever, in our opinion, that pass muster for dengue in Havana, and many other points to avoid the rigors of quarantine, on account of the mildness of the fever, or the lateness of the season in which they occur.

These cases come under Findlay's classification of acclimating or non-albuminuric yellow fever, though if you examine the urine of some of these cases two or three days after the fever has disappeared, you will very likely detect a trace.

It is our impression that the immunity claimed for children reared in tropical climates from yellow fever is due primarily to the children having actually had this mild yellow fever without the parents or the doctor recognizing same at the time of the attack.

It is particularly in this mild type of yellow fever, or dengue (as it was originally called), which preceded each outbreak or epidemic of yellow fever, in which the doctor experiences the greatest difficulty in actually saying, "This is, or is not, yellow fever."

The distinguishing points that we have to watch in these mild cases is a slight jaundice of the conjunctivȧ, even when the skin is not tinged, great prostration, out of all proportion to the mildness of the fever, rather more head and back pains than you would expect from a temperature of 100°, a slight trace of albuminuria a day or two after first onset; a progressively decreasing pulse to perhaps 50 or 60 may be reached where the pulse normally would have been 70 or 80. These cases occurring in groups in neighborhoods, all seeming to originate from some particular house, or occurring after attendance upon some funeral or sick friend, all of such environments or conditions would tend toward the establishment of a diagnosis of yellow fever. But to be absolutely positive in such cases requires more than a single visit or observation upon one case. It necessitates all the corroborative evidence to make the careful diagnostician absolutely sure of his position, before he subjects any community to the great inconvenience and even injustice of many of our modern quarantines.

While the presence of albumin in the urine is a corroborative symptom, its absence does not render it impossible to be a case of yellow fever. Where the kidney functions are active and a great deal of water has been imbibed, it is possible to suffer from a very mild case of yellow fever without albumin occurring at all.

An ordinary malarial chill grafted onto a case of intermittent albuminuria occurring during yellow fever epidemics is almost invariably reported as a genuine case of yellow fever, and this is particularly true if catarrhal jaundice happens to complicate the same case.

Albuminuria may be cyclic, dietetic, febrile, gouty, nephrogenous, false when the albumin comes from pus or secretions in the urinary passage. You will thus see some of the many conditions in which we have albuminuria that might be associated with pneumonia, tonsilitis, malaria, typhoid, influenza, dengue, or any of the infectious fevers which, grafted onto the above conditions producing albuminuria, might, at least, if occurring during the prevalence of yellow fever, cause a wrong diagnosis to be made.

It is in such conditions as these just described that puzzles the clinician when he really comes to make his diagnosis in the first cases of yellow fever occurring in a given community.

There is a very close similarity between estivo-autumnal and severe cases of yellow fever—each is ushered in with a chill, followed by high fever, albuminous urine with strong tendency to hemorrhage and congestion of internal organs. It is practically a matter of impossibility to distinguish one from the other within less than four or five days, and then the chief point of difference is in finding the crescent malarial bodies in the blood of the estivo-autumnal patient. The clinical pictures of each runs in such parallel lines that it is next to impossible to make the differential diagnosis without the aid of the microscope.

This variety of malarial fever occurs mostly in the late summer and autumn months and is occasionally designated malignant, hemorrhagic, malarial fever, swamp or rice fever, the prognosis of which is as bad as the worst type of yellow fever. Several times we have had honest difference of opinion from our expert boards as to whether a given case was yellow fever or estivo-autumnal fever.

The clinical pictures are practically the same—the finding of malarial crescents in the blood proves the case to be partly malarial, though it may be mixed infection; at the same time there are so many parallels between malarial and yellow fever, so many symptoms and conditions identical, each having the mosquito as an intermediary host, that it would not be surprising to us, if the etiological factor in yellow fever, when finally worked out, proves to be some blood parasite similar to the malaria plasmodium, that it is ultra-microscopic, or it may be possible that it is some ptomain resulting from some parasite which our microscopist, up to date, has not been able to isolate.

Yellow fever among the darkies is hard to diagnose. It is more common than generally believed, on account of their having fewer mosquito-bars to protect them from the stegomyia; their disposition to sleep at any and all times wherever night overtakes them. Their usual mosquito-netting is of wide mesh or badly torn; their desire for attending the sick, and especial pleasure in going to funerals — rendering them peculiarly liable — in thus visiting the infected house where yellow fever is not known to exist. They visit the sick, not only of their own race, but of all their neighbors, sometimes from curiosity, and again to play the part of the good Samaritan.

The difficulty of diagnosing yellow fever among the colored people is exaggerated from the fact that the absence of one of the cardinal symptoms — jaundice — except in the conjunctiva of the eye.

The stasis or congestion in the white person is the counterpart of the skin with ashy hue in the colored subject suffering from yellow fever. The fever, as a rule, does not register so high, nor is the secondary rise so apt to occur as in the Caucasian subject. The disproportion between the pulse and temperature is more marked in the colored race, perhaps, than in the white; the pulse rate of 45 to 55 is not of uncommon occurrence.

So many blue-gum negroes have naturally red or congested eyes, from the little care or protection which they have from fire, dust, and sun, and so a congested or even inflamed condition of the eye does not count as much for yellow fever in the colored race as it would in the white. After three days of the pronounced fever, close inspection will usually reveal a yellow tinge in the conjunctival portion of the eye. Many darkies, however, have this hue in perfectly normal condition.

Inflamed, bleeding gums in the darkey, would cause more suspicion of yellow fever than would the same condition in the white race, for usually their teeth are good, in cases where no salivation has previously existed, swollen, thick lips, red throat and narrow tongue, with a very irritable stomach, and the above symptoms alluded to, would present a very suspicious case of yellow fever in our brother in black.

The negro mortality is twice as great in the average diseases as in the Caucasian. In yellow fever the very reverse of the proposition is true. In fact, in the milder types of yellow fever among the colored people at Tallulah, La., Drs. Gaines and Bass are authority for the fact that many suffering with mild yellow fever did the cooking and cotton picking for the town and country around Tallulah, La., in the epidemic of 1905.

In any number of cases of fever running about the same course occurring in succession in the same family or neighborhood, show how a direct connection from one house to another,—cases of fever that usually run their course in one or two days,—in which one or two doses of quinin seem to effect a cure, but who, in reality, would recover just as quickly without it,—cases in which there is a disproportion between the temperature of the body and the great weakness which follows the attack.

Be especially suspicious of cases which fall sick from three to five days after visiting some house or funeral, or in cases that have developed in from fifteen to eighteen days after some sick stranger has perhaps visited a neighborhood.

In yellow fever times, particularly, inspect any large number of reported deaths from uremia, acute parenchymatous hepatitis, Bright's disease, bilious remittent fever, swamp or hemorrhagic malarial, typhoid fever, broncho-pneumonia, and tonsilitis with auto-infection, as any of these diseases may be confounded with yellow fever in those who are inexperienced in its diagnosis and management.

In the diagnosing of yellow fever in any given community it would be quite a help in coming to a conclusion if the stegomyia fasciata mosquito was found to be coexistent with the fever; while not being able to find stegomyia would not prove absence of yellow fever, it would be a strong corroborating fact should stegomyia be plentiful in the community.

We must remember in differentiating yellow fever from other conditions that in an uncomplicated case of yellow fever we would have the following clinical picture:

Sudden onset, usually at night, severe lumbar, head and leg pains, peculiar facies with anxious expression and congested eyes, pulse bounding and full, especially as temperature rises; usually nausea and vomiting of bile, pain and tenderness over the stomach, intense capillary congestion of the surface of the body, hyperemia, which disappears on pressure of the finger, but quickly returns when pressure is relieved; usually mental dullness and greater prostration than you would expect in ordinary fever.

This clinical picture may last for twenty-four hours, when it may be followed by lysis, or secondary rise in temperature, or collapse, jaundice, black vomit, anuria with hemorrhage from gums, nose, vagina, and rectum, as well as the black vomit of the stomach, which is considered characteristic of the disease. Hyperemia, herpes-labialis are not the rule in unmixed yellow fever.

---

TULANE.—At the seventy-second annual commencement, May 2, 1906, the Medical Department of Tulane University graduated 119 students: 102 in medicine and 17 in pharmacy. Surgeon J. H. White, M. H. S., delivered the annual address.

---

DR. JOSEPH GILBERT, of Austin, County and City Health Officer, has been appointed physician to the A. and M. College of Texas, Bryan, Texas, vice Dr. Lanham, son of the Governor, resigned. The Austin people, while congratulating Dr. Gilbert, regret to part with so able and efficient a health officer. Dr. Gilbert is a graduate of the A. and M., as well as of the Medical Department of the University of Texas at Galveston. Dr. J. M. Loving was elected to succeed Dr. Gilbert.

